# Acute changes in liver tumour perfusion measured non-invasively with arterial spin labelling

**DOI:** 10.1038/bjc.2016.51

**Published:** 2016-03-31

**Authors:** S Peter Johnson, Rajiv Ramasawmy, Adrienne E Campbell-Washburn, Jack A Wells, Mathew Robson, Vineeth Rajkumar, Mark F Lythgoe, R Barbara Pedley, Simon Walker-Samuel

**Affiliations:** 1 UCL Cancer Institute, University College London, Paul O'Gorman Building, 72 Huntley Street, London WC1E 6DD, UK; 2 UCL Centre for Advanced Biomedical Imaging, University College London, Paul O'Gorman Building, 72 Huntley Street, London WC1E 6DD, UK; 3 Cardiovascular and Pulmonary Branch, Division of Intramural Research, National Heart, Lung and Blood Institute, National Institutes of Health, Bethesda, MD 20892, USA

**Keywords:** perfusion, MRI, liver, cancer, metastasis, vascular disrupting agents

## Abstract

**Background:**

Non-invasive measures of tumour vascular perfusion are desirable, in order to assess response to vascular targeting (or modifying) therapies. In this study, hepatic arterial spin labelling (ASL) magnetic resonance imaging (MRI) was investigated to measure acute changes in perfusion of colorectal cancer in the liver, in response to vascular disruption therapy with OXi4503.

**Methods:**

SW1222 and LS174T tumours were established in the liver of MF1 nu/nu mice via intrasplenic injection. Perfusion and *R*_2_^*^ MRI measurements were acquired with an Agilent 9.4T horizontal bore scanner, before and at 90 min after 40 mg kg^−1^ OXi4503.

**Results:**

A significant decrease in SW1222 tumour perfusion was observed (−43±33%, *P*<0.005). LS174T tumours had a significantly lower baseline level of perfusion. Intrinsic susceptibility MRI showed a significant increase in *R*_2_^*^ in LS174T tumours (28±25%, *P*<0.05). An association was found between the change in tumour perfusion and the proximity to large vessels, with pre-treatment blood flow predictive of subsequent response. Histological evaluation confirmed the onset of necrosis and evidence of heterogeneous response between tumour deposits.

**Conclusions:**

Hepatic ASL-MRI can detect acute response to targeted tumour vascular disruption entirely non-invasively. Hepatic ASL of liver tumours has potential for use in a clinical setting.

Angiogenesis is a key hallmark of cancer biology (Hanahan and Weinberg, 2011), and while this enables the delivery of nutrients and oxygen to meet the metabolic demands of the malignant cells, it also provides a route through which to administer therapeutic agents. Methods for measuring tumour vascular perfusion are therefore in demand, both as research tools and as a means to characterise and monitor tumours in patients. The development of non-invasive measures of vascular perfusion in tumours is particularly appropriate as the conventional radiological assessment of response (using the RECIST criteria; Eisenhauer *et al*, 2009) evaluates changes in tumour size, which overlooks functional changes that can occur over much shorter timescales and could be equally indicative of a successful response to the therapy.

A number of magnetic resonance imaging (MRI) techniques have been used to assess acute response to vascular targeted therapy, including contrast-enhanced MRI (Patterson *et al*, 2012b), intrinsic susceptibility (IS)-MRI (Robinson *et al*, 2005), vessel size imaging (Burrell *et al*, 2012; Nielsen *et al*, 2012) and diffusion MRI (Koh *et al*, 2009; Nathan *et al*, 2012). However, the baseline perfusion of the tumour vasculature, and the acute (i.e., within several hours of administration) assessment of response to vascular therapy could provide a better understanding of the tumour vascular microenvironment and potentially predictive indicators of drug action. Intrinsic susceptibility MRI has shown promise in the acute setting, with some indication of prognostic value in pre-therapy measurements (Robinson *et al*, 2005). The IS-MRI can be used to quantify deoxyhaemoglobin build-up via assessment of the transverse relaxation rate *R*_2_* however, physiological interpretation of variations in such measurements can be challenging due to its dependence on numerous physiological processes, such as vascular geometry, paramagnetic cellular debris associated with necrosis, and haemosiderin. A direct method for measurement of tumour perfusion would therefore be an improvement on IS-MRI-measured vascular haemodynamics.

We recently investigated the use of arterial spin labelling (ASL) MRI to measure the combined arterial and portal venous perfusion in both normal mouse liver and a mouse model of colorectal metastasis to the liver (Ramasawmy *et al*, 2014). ASL uses the flow of blood water to estimate perfusion in ml g^−1^ min^−1^, and thus does not require administration of a contrast agent, allowing for repeated perfusion measurements without the need to wait for a contrast agent to disperse. Perfusion can then be measured before and immediately following drug administration. Moreover, hepatic ASL offers good potential for translation onto human MRI scanners and for use in the clinic in these deep-sited tumours, particularly for patient populations that cannot be administered a contrast agent, as it has been employed in other liver conditions (Bokkers *et al*, 2012; Zou *et al*, 2014).

In the current study, we have continued our previous work by investigating the utility of ASL in the assessment of acute response to the second-generation combretastatin molecule OXi4503 (combretastatin A1 di-phosphate/CA1P), a potent vascular disrupting agent, in a mouse model of colorectal metastasis to the liver. Two human colorectal carcinoma cell lines were studied (LS174T and SW1222), with well-characterised and markedly different vascular characteristics (El *et al*, 2007). The aim of this research was to evaluate whether ASL can provide a direct measure of tumour perfusion for prognostic measurements both before and at an acute time point after vascular targeted therapy. As the model used created numerous metastatic tumours within the liver, a secondary aim of the study was to investigate the relationship between response to therapy and the location of tumour deposits within the liver.

## Materials and methods

### Cell lines and tissue culture

The human colorectal carcinoma cell lines SW1222 and LS174T (El *et al*, 2007) were cultured in Dulbecco's Modified Eagles Medium (PAA, GE Healthcare, Fairfield, CT, USA) containing 5 mM L-glutamine (PAA, GE Healthcare) and 10% v/v Fetal bovine serum (Life Technologies, Carlsbad, CA, USA). Cells were grown in a 37 °C humidified incubator at 5% CO_2_.

### *In vivo* model of liver metastasis

All animal studies were approved by the University College London Biological Services Ethical Review Committee and licensed under the UK Home Office regulations and the Guidance for the Operation of Animals (Scientific Procedures) Act 1986 (Home Office, London, UK) and United Kingdom Co-ordinating Committee on Cancer Research Guidelines for the Welfare and Use of Animals in Cancer Research (Workman *et al*, 2010). To establish liver tumours (Fidarova *et al*, 2008), MF1 *nu/nu* mice (female, 6–8 weeks old, 25–30 g) were anaesthetised with isoflurane in O_2_ at a concentration of 4% for induction, followed by 1.5% for maintenance. A subcutaneous injection of 0.1 mg kg^−1^ buprenorphine (Vetergesic, Reckitt Benckiser, UK) was administered and the flank of the animal sterilised with clorhexidine solution. A laparotic incision of approximately 1 cm was then made above the location of the spleen and the organ exteriorised for injection. SW1222 (*n*=6) or LS174T (*n*=6) cells were injected intrasplenically at a concentration of 1 × 10^6^ cells in 100 *μ*l of serum-free medium and allowed to wash through to the liver for 5 min, followed by splenectomy to avoid the development of tumours at the site of injection. The laparotic incision was sutured (Ethicon, Johnson & Johnson, New Brunswick, NJ, USA) and closed with wound clips (Autoclip system; Harvard Apparatus, Cambridge, UK).

### Magnetic resonance imaging

All MRI was performed on a 9.4-T Agilent VNMRS scanner (Agilent Technologies, Santa Clara, CA, USA) with a 39-mm birdcage coil (RAPID Biomed, Rimpar, Germany). Mice were anaesthetised with isoflurane in O_2_ at a concentration of 4% for induction followed by 1–2% for maintenance. Mice were then positioned in the centre of the bore of the magnet, maintained at 37 °C with heated water pipes, and monitored using respiratory bellows and a rectal temperature probe (SA Instruments, Stony Brook, NY, USA).

### Assessment of model development with morphological MRI

A respiratory-gated, high-resolution, *T*_2_-weighted, fast spin echo (FSE) sequence was used to determine tumour burden at 3, 4 and 5 weeks post implantation and later used to classify the location of tumour deposits. Sequence parameters included: field of view (FOV)=35 × 35 mm^2^, matrix size=256 × 256, TR=1500 ms, echo train length=4, effective TE=26 ms, 40 × 0.5 mm contiguous slices to cover the entire liver, and four averages.

Voxels corresponding to liver and tumour tissue, which appeared hyper-intense relative to normal liver, were manually segmented using Amira (Visualisation Sciences Group, Hillsboro, OR, USA). Tumour locations were manually assessed on high-resolution *T*_2_ weighted images and divided into three categories: (a) peripheral, (b) near small, and (c) near large vessels. The limit to the tumours' proximity to major vasculature was defined as 3–4 voxels (0.5 mm). Venous and arterial blood vessels appear hypo-intense on the *T*_2_-weighted image and small vessels were categorised by a cross-sectional area less than 0.1 mm^2^ (approximately 7–8 voxels in diameter).

### Functional assessment of liver tumour response to vascular disruption

Assessment of vascular perfusion change within liver tumours following therapy with the VDA OXi4503 was performed at 5 weeks post surgery. All six SW1222 tumour-bearing mice were dosed with 40 mg kg^−1^ of OXi4503, with normal liver acting as an inherent internal control in order to minimise animal usage and to provide relevant indication of functional change within the tumours. Only three out of six LS174T models developed distinct liver tumours; therefore, only these were put forward for VDA therapy, again using internal controls of normal-appearing liver tissue. Based on results from these initial experiments, a further six SW1222 models were set up as external standalone controls, five of which produced liver metastases. These mice were set up and imaged exactly as the treated experimental groups albeit dosed with a saline sham dose instead of OXi4503.

A primed intravenous (i.v.) line was positioned in the tail for administration of OXi4503. Following morphological scanning, an axial slice through the liver was identified for ASL and IS-MRI scans. Baseline perfusion and *R*_2_* measurements were performed before administering 40 mg kg^−1^ OXi4503 (4 mg ml^−1^ stock solution injected at 10 ml kg^−1^ of body weight) via the i.v. line. *R*_2_* measurements were acquired dynamically up to 90 min post dose, and were followed by a second perfusion acquisition. The time point of 90 min was used as previous in-house experiments in subcutaneous tumours showed *R*_2_* increases after OXi4503 dosing within this time frame.

### Arterial spin labelling

Arterial spin labelling data were acquired using a single-slice respiratory triggered, Look-Locker flow-sensitive alternating inversion recovery (FAIR) with a segmented gradient-echo readout (Campbell-Washburn *et al*, 2013). Sequence parameters included: slice thickness=1 mm, FOV=30 × 30 mm, matrix size=128 × 128, echo time (TE)=1.18 ms, inversion time spacing=110 ms, Look-Locker readout pulse spacing 2.3 ms, flip angle=8°. Sequence repetition time: 13 s, 50 inversion recovery readouts, 4 lines per segmented acquisition. A 6-mm localised selective inversion was followed by a global inversion, with the whole scan lasting around 14 min.

Total liver perfusion was quantified using methods previously described (Ramasawmy *et al*, 2014); briefly, the data were quantified with the Belle model (Belle *et al*, 1998), alongside an intravascular capillary blood longitudinal relaxation time that had been previously measured in the ventricular blood pool of the mouse heart at 1.9 s (Campbell-Washburn *et al*, 2012). A blood-tissue partition coefficient of 0.95 ml g^−1^ was taken from ^85^Krypton gas-clearance measurements previously reported (Rice *et al*, 1977), and this constant was used for both liver and tumour regions. A three-parameter fit of the inversion recovery, corrected for the Look-Locker signal saturation (Deichmann, 1992), was used for longitudinal relaxation time (*T*_1_) quantification following retrospective gating analysis.

Perfusion values were calculated in regions of interest (ROIs) corresponding to either tumour or normal tissue in the single slice acquired in the ASL acquisition. Mean perfusion values were estimated in each ROI.

### Intrinsic susceptibility MRI

Values of the transverse relaxation rate (*R*_2_*) were estimated from a respiratory-gated multi-echo, multi-slice gradient-echo sequence (MGEMS). Sequence parameters included: 8 echoes (TE_1_=2 ms, echo spacing=2 ms), TR=280 ms, matrix size=128 × 128, FOV=30 × 30 mm^2^, 15 slices, and slice thickness=1 mm. *R*_2_* was estimated using a maximum likelihood approach that took into account the Rician distribution of the data (Walker-Samuel *et al*, 2010). Regions of interest corresponding to individual tumours were manually defined on all slices within the liver, and the average *R*_2_* value estimated in each tumour.

Wilcoxon tests were performed on metastases and internal controls of normal-appearing liver, before and after *in situ* dose for both perfusion and *R*_2_* measurements.

### Histological assessment

Tumours were excised at 24 h post VDA administration for histological assessment. Animals were administered 12.5 mg kg^−1^ of the perfusion marker Hoechst 33342 i.v. 1 min before culling. Livers were excised and snap frozen in isopentane/liquid nitrogen, before sectioning on a cryostat at 10 *μ*m thickness. Immunohistochemical (IHC) staining for the blood vessel marker CD31 was performed before imaging on a fluorescence microscope (AxioImager, Zeiss, Oberkochen, Germany). Haematoxylin and eosin staining for basic cell morphology was performed and imaged on a brightfield microscope (AxioSkop, Zeiss).

## Results

### SW1222 *vs* LS174T perfusion measurements

**Figure 1:**
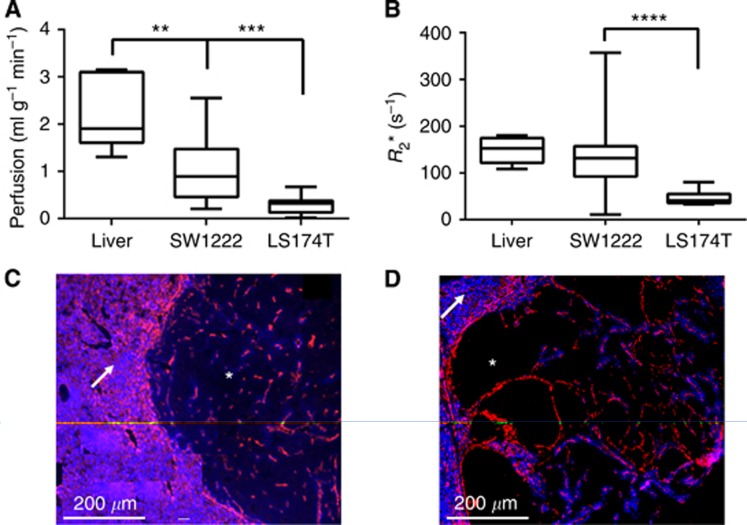
**Baseline perfusion of tumour metastases.** Average baseline estimates of blood perfusion from ASL (**A**) and *R*_2_* from IS-MRI (**B**) in normal-appearing liver (*n*=6), SW1222 tumours (*n*=6) and LS174T tumours (*n*=3) (** indicates *P*<0.01, *** indicates *P*<0.005, **** indicates *P*<0.001 significant differences). Fluorescence microscopy of blood vessels (CD31, red) and perfusion (Hoechst 33342, blue) in SW1222 tumours (**C**) and LS174T tumours (**D**). Arrows indicate areas of normal liver tissue and stars indicate areas of tumour tissue.

Initial measurements of perfusion and *R*_2_*, for both SW1222 and LS174T metastases and normal surrounding liver tissue (at 5 weeks post implantation), can be seen in Figure 1. Segmentation of the metastases was performed based on *T*_1,selective_ maps, as it was observed that the tumour *T*_1,selective_ was significantly greater (*P*<0.0001, Kruskal–Wallis ANOVA) than that of the liver and major vasculature for both cell lines: *T*_1,liver_ 1.34±0.07 s (mean±s.d., data combined from both cell lines), *T*_1,Portal Vein_ 0.29±0.21 s (both cell lines), *T*_1,SW122_ 2.15±0.11 s (*n*=6), and *T*_1,LS174T_ 2.49±0.13 s (*n*=3).

Perfusion measurements from these metastases showed a significant difference (*P*<0.0001, Kruskal–Wallis ANOVA) between the liver, SW1222 and LS174T metastases: *P*_liver_ 2.32±0.34 ml g^−1^ min^−1^ (mean±std), *P*_SW1222_ 1.03±0.72 ml g^−1^ min^−1^, *P*_LS174T_ 0.28±0.16 ml g^−1^ min^−1^ (Figure 1A). This lower level of perfusion in tumours, compared with normal liver, is consistent with qualitative fluorescence microscopy imaging of perfusion seen in Figure 1C and D, where the presence of the perfusion marker Hoechst (blue) is lower in the tumour tissue (*) for both SW1222 (Figure 1C) and LS174T (Figure 1D) compared with normal surrounding liver parenchyma (arrow). Blood vessels, marked by CD31 in red, show a decreased abundance, penetration, and organisation within tumour tissue compared with normal liver.

A difference between SW1222 and LS174T tumours is also observable, with fewer blood vessels and perfusion in the LS174T tumours, further corroborating the obtained ASL measurements. This may contribute to the observed difference in *R*_2_* (Figure 1B), where LS174T tumours have a significantly slower value than SW1222 tumours and normal liver. A significant difference (*P*<0.0001, Kruskal–Wallis ANOVA) was measured between average *R*_2_* estimates in normal-appearing liver, LS174T tumours and SW1222 tumours: *R*_2_*_liver_=148±010 s^−1^, *R*_2_*_SW1222_=144±17 s^−1^, and *R*_2_*_LS174T_=47±4 s^−1^.

### Functional response of the tumour vasculature to disruption with OXi4503

**Figure 2:**
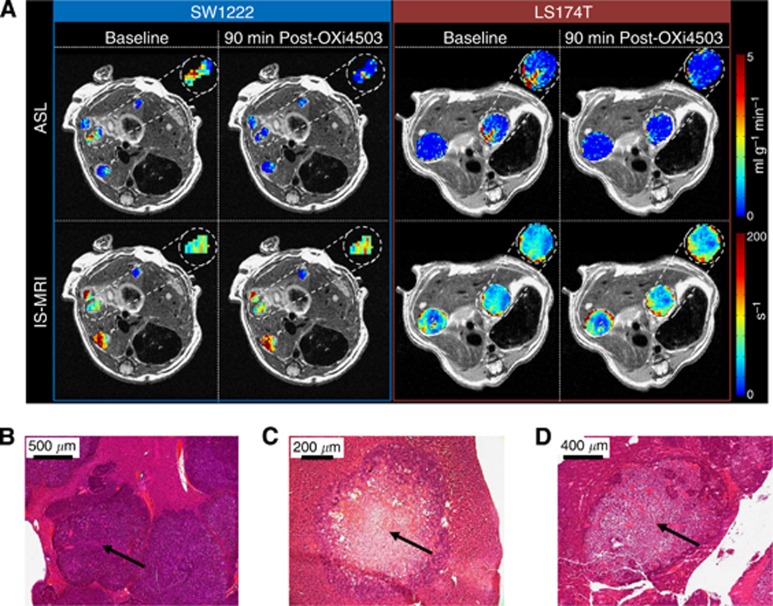
**Functional response of the tumour vasculature to disruption with OXi4503.** Example high-resolution, axial images (**A**) with perfusion (top row) and *R*_2_* (bottom row) maps overlaid on tumour ROIs (SW1222 on the left, and LS174T on the right, pre- and post OXi4503 therapy). A heterogeneous spatial distribution can be seen in both parameters, both within individual tumour deposits and across different tumours within the same liver. Histological staining by H&E is shown for saline control (**B**), and OXi4503-dosed SW1222 (**C**) and LS174T (**D**) tumours. The arrows highlight the centre of liver tumour deposits (**B**, viable and **C** and **D**, necrotic).

Before OXi4503 delivery, tumours displayed variable baseline measurements of perfusion and *R*_2_*, with a heterogeneous spatial distribution for each parameter within each tumour. Following treatment with OXi4503, a heterogeneous response was observed in both tumour types by ASL and IS-MRI techniques, as can be seen in the example maps shown in Figure 2A. Absolute and relative changes from baseline in perfusion and *R*_2_* measurements across groups are shown in Table 1, with normal appearing liver from the same animals included as an internal control; a significant decrease in perfusion was detected in SW1222 metastases for both treated and saline control groups. The decrease in perfusion of the saline control group is not fully understood, though actual perfusion change is small, it is potentially due to minor dehydration caused by prolonged imaging (Ramasawmy *et al*, 2014). No significant change was measured in treated LS174T tumours, probably due to their low levels of baseline perfusion. Perfusion in normal-appearing liver tissue was varied, although it decreased non-significantly across the groups.

For change in *R*_2_* the only significant change measured was within the VDA-treated LS174T tumours, with no significant changes observed in the SW1222 tumours (treated and control) or normal-appearing liver. Histological assessment of tumour metastases viability by H&E staining shows that saline-dosed control SW1222 tumours (Figure 2B) are highly nucleated and viable, with little areas of necrosis, while both OXi4503-treated SW1222 (Figure 2C) and LS174T (Figure 2D) tumours have large central necrosis with a small rim of surviving tumour cells surrounding the metastasis. No change was observed in the surrounding normal liver tissue.

### Relationship between baseline and post-therapy parameter estimates

**Figure 3:**
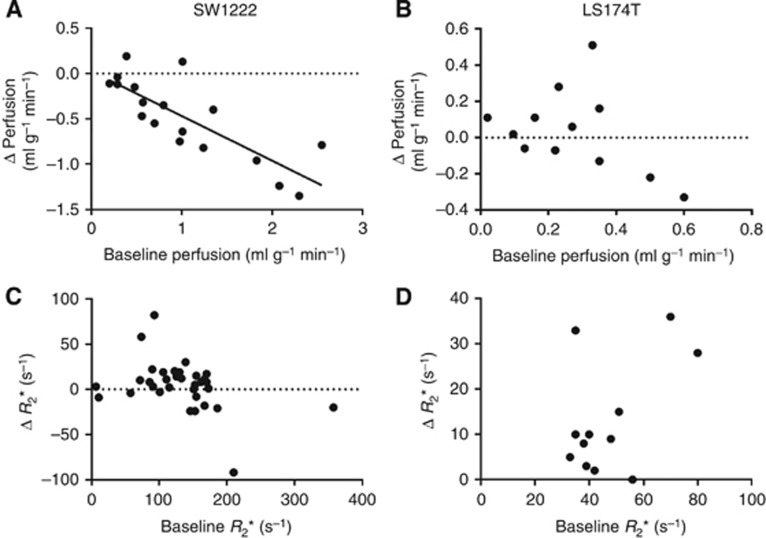
Correlation of baseline and post-OXi4503 measurements for perfusion (top row) and *R*_2_* (bottom row). Graphs show the change in the value of each parameter in individual tumour deposits *vs* the baseline value. The change in perfusion measured in SW1222 tumours (**A**) displayed a significant negative correlation with baseline measurements (*R*^2^=0.76, *P*<0.001 Pearson's *R*). Better-perfused tumours at baseline exhibited a greater loss of perfusion following OXi4503 administration. LS174T tumour perfusion (**B**) was low at baseline, and no similar trend was found. For both SW1222 (**C**) and LS174T (**D**) tumours, the *R*_2_* baseline data showed no correlation with baseline values.

Figure 3 displays the results of an analysis comparing the change in perfusion and *R*_2_* values at 90 min following Oxi4503 administration, with values measured at baseline. A significant, negative correlation (*R*^2^=0.76, *P*<0.001 Pearson's *R*) was found between the change in tumour perfusion and the value at baseline in SW1222 tumours (Figure 3A). However, no corresponding correlation was observed between baseline *R*_2_* and subsequent change in IS (*P*>0.1, see Figure 3C). For LS174T tumours, no significant correlation (*P*>0.1) was measured between the 90-min changes in perfusion and baseline perfusion (Figure 3B), nor between baseline and 90 min changes in *R*_2_* (Figure 3D).

### Relationship between response to OXi4503 and tumour location

**Figure 4:**
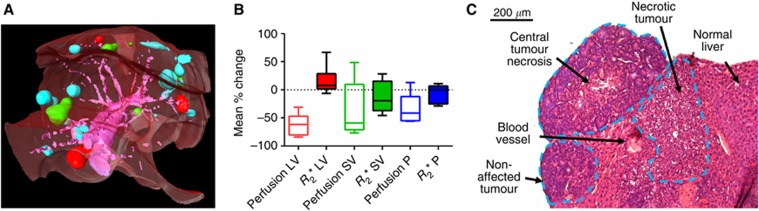
**The dependency of tumour location within the liver and the magnitude of perfusion changes.** Analysis of SW1222 tumours following dosing with OXi4503. (**A**) A three-dimensional visualisation of an example mouse liver at week 5 post implantation, showing liver parenchyma (semi-transparent red), and major vasculature (purple). Tumours have been coloured according to their proximity to either a large vessel (>0.1 mm^2^, red), small vessel (green) or no visible vessel (blue). (**B**) The percentage change in perfusion and *R*_2_* at 90 min following administration of 40 mg kg^−1^ of OXi4503, categorised according to proximity to vessels and location within the liver. Large perfusion decreases were observed in tumours close to either large (LV) or small (SV) blood vessels, although tumours near smaller vessels demonstrated a more variable response (as evidenced by larger error bars). Tumours at the periphery (P) of the liver did not display a significant change in perfusion. No significant changes were measured in *R*_2_* in any of the liver locations. (**C**) Example H&E slice of liver containing three different tumours located at a range of position to a blood vessel, demonstrating a heterogeneous response to OXi4503 within the same liver.

OXi4503-treated SW1222 tumours were categorised to evaluate the effect of location within the liver and their subsequent therapeutic response. Figure 4A shows a manually-segmented three-dimensional rendering of a mouse liver from *T*_2_-weighted MRI data, in which the liver parenchyma can be seen in semi-transparent red, the major vasculature in purple. Tumours have been coloured as classified by their proximity to large vessels (>8 voxels, red), small vessels (<8 voxels, green) and distal (>4 voxels, blue) to vasculature – typically located at the periphery of the liver lobe (blue).

Figure 4B shows the change in perfusion (red, green and blue outline) and *R*_2_* (solid red, green and blue) at 90 min post OXi4503 when liver tumours were grouped according to their location (LV: large vessels, SV: small vessels, and P: peripheral). Tumours close to major vasculature were better perfused at baseline (*P*<0.05, Kruskal–Wallis ANOVA): 1.5±0.8 ml g^−1^ min^−1^ (mean±standard deviation), 1.3±0.7 ml g^−1^ min^−1^, 0.6±0.3 ml g^−1^ min^−1^ for locations LV, SV, and P, respectively. No significant differences in perfusion reduction were measured between the groups (*P*>0.1, Kruskal–Wallis ANOVA), though the data suggest that the largest perfusion decreases can be observed in metastases proximal to vasculature. The most consistent mean decrease in perfusion was observed close to large vessels (−62±19%, mean±standard deviation), whereas a more varied decrease was seen in tumours near smaller vessels (−36±51%). Tumours in the periphery of the liver lobe displayed a smaller but highly variable response in perfusion (−32±25%).

No significant differences (*P*>0.05, Kruskal–Wallis ANOVA) were measured between baseline *R*_2_* measurements, which were 110±50 s^−1^, 270±140 ms^−1^, and 210±110 s^−1^ for locations LV, SV, and P, respectively. No significant differences were measured in *R*_2_* response post-OXi3403 as grouped by location (*P*>0.15, Kruskal–Wallis). Metastases in all locations behaved inconsistently for *R*_2_* (large vessel 16±26% (mean±standard deviation), small vessels −13±29%, and periphery −4±20%). Histological assessment (Figure 4C), while not quantitative, provided some evidence that tumour deposits located in close proximity could have different responses to treatment, potentially moderated by their distance from major blood vessels.

## Discussion

The aim of this study was to evaluate the response of a model of liver metastasis to vascular disruption using hepatic ASL-MRI to measure tumour perfusion, alongside IS-MRI as a measure of deoxyhaemoglobin accumulation. This is a novel application of ASL, demonstrating the first use of hepatic ASL-MRI to assess response to vascular targeting therapy within the liver. The results presented in this paper are consistent with previous research using ultrasound imaging in subcutaneous tumours imaged before OXi4503 treatment and 30 min after (Mason *et al*, 2011), previous acute studies of a different VDA (ZD6126) (Robinson *et al*, 2005), the proposed vascular targeting action of the agent (Siemann, 2011), and relate well to *ex vivo* histological data from a similar VDA (CA4P) used in SW1222 subcutaneous tumours (El-Emir *et al*, 2005). The results are also consistent with other studies investigating OXi4503 (CA1P) in particular, which has been shown to significantly reduce tumour blood vessel perfusion within 30 min of dosing with a 90% reduction observed at 4 h, albeit in a subcutaneous setting of different tumour types (Salmon and Siemann, 2006), using *ex vivo* histology and *in vivo* dynamic contrast enhanced MRI. OXi4503 has also been used in an intravital setting through use of a window chamber model of breast cancer (Wankhede *et al*, 2010), showing tumour vascular structural collapse from the earliest time-point imaged of 2 h.

The use of hepatic ASL in the present study allowed novel insight into the dynamics of response to vascular disruption in an orthotopic setting and exogenous contrast label free, based on tumour pathophysiology and location within the liver. These observations could potentially inform future prognostic markers of response, and are discussed below.

### Hepatic ASL is able to monitor differences in response to vascular disruption

Given that the extent of vascularisation of LS174T tumours is known to be low and much less extensive than the SW1222 tumours (Kalber *et al*, 2008; Folarin *et al*, 2010), which in turn are less well perfused than normal tissue (Figure 1), the perfusion measured in these different tissues reflect this. A significant reduction in perfusion was observed at 90 min following administration of OXi4503 in SW1222 tumours, while perfusion changes in LS174T tumours were highly varied, due to the low baseline values (Figure 2A; Table 1). A low initial level of perfusion is a limitation of the ASL technique's sensitivity, as further decreases may be below the detection threshold of the technique, though this would indicate that there was minimal vasculature to target. A reduction in perfusion of SW1222 tumours was also noted after saline control dose, albeit not as pronounced; the physiological mechanism behind this change is not known; however, it does highlight the sensitivity of the method used.

A significant change in *R*_2_* measurements was only observed in post-treatment LS174T tumours, indicating that a build-up of deoxyhaemoglobin and subsequent change in *R*_2_* may act as a complimentary biomarker when tumour perfusion is minimal. Histological assessment of tumour deposits showed viable tissue for saline controls (Figure 2B), and centrally necrotic deposits for OXi4503-treated SW1222 (Figure 2C) and LS174T (Figure 2D) tumour tissue.

### Relationship between baseline and post-OXi4503 perfusion measurements

We observed that baseline perfusion in SW1222 tumour deposits was significantly correlated with the subsequent decrease in perfusion at 90 min following OXi4503 administration (Figure 3). Such acute, non-invasive measurements of perfusion changes are not generally possible, as the majority of other approaches require a contrast agent to be administered (Lee *et al*, 2014), and disperse, before follow-up measurements can be taken. One of the few examples of rapid tumour imaging of perfusion without the need for extended periods of contrast agent dispersal is the use of microbubbles in ultrasound, which are dispersed within minutes following administration (Mason *et al*, 2011). Ultrasound has also been shown to be capable of imaging vascular disruption over a similar time course without contrast agent, through the use of Colour-Doppler technique (Hadimani *et al*, 2013). However, this was in a superficial subcutaneous tumour siting; in this study, we have shown that ASL-MRI can correlate pre- and post-treatment perfusion in tumour sited deeply within an organ.

Therefore, ASL-MRI could be a predictor of acute VDA efficacy, with potential use as a diagnostic tool for patient stratification and treatment planning. *R*_2_*, for example, has been used clinically to assess response to OXi4503, though no acute response (<3 h) was seen (Patterson *et al*, 2012a). The potential to highlight those metastases that may respond positively to treatment *vs* those that will not, based on baseline perfusion, could spare patients from unnecessary treatment. Targeting of different tumour pathophysiologies often requires combination approaches to treatment for adequate response, especially with regard to VDAs (Pedley *et al*, 2002), so knowledge obtained from a biomarker of tumour perfusion could provide a deeper insight into the mechanics of tumour response to therapy *in vivo*.

Robinson *et al* (2005) found baseline *R*_2_* to be predictive of subsequent response, although this was evaluated in different cell lines and using a different VDA, and in a different tumour model. No equivalent result was found in this study, which could be due to the difficulty in measuring *R*_2_* in liver, particularly as shimming of the *B*_0_ field can be challenging due to susceptibility artefacts propagating from surrounding lungs and gut, in addition to respiratory and peristaltic motion artefacts which can be difficult to control.

### Heterogeneity of response to therapy based on tumour location

Vascular disrupting agents are often said to be more effective against larger tumours (Landuyt *et al*, 2000; Liu *et al*, 2015); however, in this study we found no obvious dependence of change in perfusion based on size. Certain tumour deposits, however, based on histological assessment, were not affected by OXi4503 at 24 h post treatment (Figure 4C). As this was an unexpected result, it was not possible to retrospectively link affected tumours to perfusion measurements. The noted heterogeneity could be a function of the time course of tumour necrosis, with those tumour deposits that had not initially responded proceeding to do so at a later point in time.

However, a potential factor influencing heterogeneous response to OXi4503 treatment is the location of the tumour deposits within the liver. The pharmacokinetics of drug delivery to the tumours might be affected by systemic perfusion within the liver. By categorising location within the liver relative to major blood vessels, in an attempt to account for different degrees of perfusion, we found that the reduction in perfusion was greatest and most consistent in metastases close to large vessels (measured to have a high level of baseline perfusion), similar but less consistent in metastases close to smaller vessels (considered to have a medium level of baseline perfusion), and was smallest in those at the periphery of the liver (considered to be the least perfused tumours) (Figure 4B).

Although we were unable to use histology data to directly link this reduction in perfusion to direct action of vascular disruption, it is still indicative of differential responses of metastases to the VDA, as also demonstrated by the range of efficacy seen in neighbouring tumour deposits by IHC (Figure 4C). This suggests that the pharmacokinetics of drug action may be influenced by micro-environmental factors such as tumour perfusion. This range of responses between metastases dependent upon location and extent of growth has been previously observed with the use of radiolabelled antibodies (Siemann, 2011), which lends support to our observations.

A limitation of this study was the use of a single-slice ASL acquisition sequence. This permitted only partial coverage of the liver; and hence, a number of metastases were omitted from analysis; longitudinal measurements were also difficult as the replication of exact image slice positions between sessions was challenging. Since completion of this study, a multi-slice adaption of the Look-Locker FAIR ASL sequence has been developed (Campbell-Washburn *et al*, 2013). Utilising the vessel-selective pseudo-continuous ASL sequences (Dai *et al*, 2008) could provide a direct method for separating the arterial and venous contributions to the liver blood supply, from which response biomarkers such as the hepatic perfusion index, a measure of the relative contribution of the two supplies in individual metastases, could be evaluated to further probe tumour pathophysiology (Cuenod *et al*, 2001; Schalkx *et al*, 2014).

## Conclusion

This study has demonstrated the use of hepatic ASL-MRI to detect acute response to the VDA OXi4503 in a mouse model of colorectal carcinoma metastasis to the liver. Significant decreases in SW1222 tumour (but not normal liver) perfusion were observed at 90 min following administration, and baseline perfusion measurements were found to be predictive of subsequent response. Moreover, response to the drug was heterogeneous between tumour deposits, with the siting of tumour deposits in the liver relative to major vessels found to have a role. Given the ability of hepatic ASL to measure acute changes in liver perfusion entirely noninvasively, it has the potential to be used in a therapeutic clinical setting.

## Figures and Tables

**Table 1:** Absolute and percentage changes (mean±s.d.) in perfusion and *R*_2_* for SW1222 metastases treated with OXi4503 (*n*=6), LS174T metastases treated with OXi4503 (*n*=3) and SW1222 metastases given saline control (*n*=5)

	**SW1222+OXi4503**	**LS174T+OXi4503**	**SW1222+Saline**
**Perfusion**			
Tumours (ml g^−1^ min^−1^)	−0.49±0.44	0.04±0.23	−0.17±0.31
%	−43±33***	61±152	−16±39*
Liver (ml g^−1^ min^−1^)	−0.35±0.28	−0.14±0.45	−0.54±0.28
%	−12±12	−10±27	−26±15
***R***_2_*			
Tumours (s^−1^)	5±28	13±12	11±16
%	0±26	28±25***	6±10
Liver (s^−1^)	12±10	16±10	8±12
%	9±11	13±11	5±7

The internal control of the liver has been included for each group. Stars denote the significance determined by Wilcoxon tests (*P<0.05, **P<0.01, ***P<0.001).
